# Deletion of the *Salmonella* pathogenicity island 2 gene, *spiC*, in attenuated *Salmonella* Typhimurium VNP20009 optimizes its potential for bacterial schwannoma therapy

**DOI:** 10.1128/mbio.01275-26

**Published:** 2026-08-10

**Authors:** Giulia Oliva, Sherif Ahmed, Manlin Shao, John J. Mekalanos, Gary J. Brenner

**Affiliations:** 1 Department of Anesthesiology, Critical Care, and Pain Medicine, Massachusetts General Hospital, Harvard Medical School 1811 , Boston, Massachusetts, USA; 2 Department of Microbiology, Harvard Medical School 1811 , Boston, Massachusetts, USA; Universiteit Gent, Gent, Belgium

**Keywords:** *Salmonella* Typhimurium, bacterial immunotherapy, NF2-related schwannomatosis, schwannoma, tumor therapy, adjuvent, tumor microenvironment

## Abstract

Recent advances in systems biology and immunotherapy have spurred the investigation of bacteria as therapeutic vehicles for cancer treatment. Currently, Bacillus Calmette–Guérin remains the only FDA-approved bacterial cancer therapy; it is a live attenuated mycobacterium that is indicated for the treatment and prophylaxis of carcinoma *in situ* of the urinary bladder and for the prophylaxis of primary or recurrent papillary tumors following transurethral resection. Although safety concerns have been raised, attenuated *Salmonella* Typhimurium strains such as VNP20009 have advanced to clinical trials targeting fast-growing human tumors. Notably, this strain induces robust immunological control of slow-growing tumors such as NF2-related schwannomatosis (NF2-SWN) in preclinical murine models. Here, we genetically characterize VNP20009 with the goal of constructing genetically defined attenuated strains that retain its promising therapeutic features while improving safety. Specifically, we investigated the contribution of the *Salmonella* pathogenicity island I (SPI-1) and SPI-2 type III secretion systems to antitumor efficacy and biosafety. Mutation of the SPI-1 gene *sipB*, a key structural component required for SPI-1 type III secretion system function, partially reduced tumor control in NF2-SWN murine schwannoma models, suggesting that bacterial invasion alone does not fully account for antitumor activity. In contrast, deletion of the SPI-2 gene *spiC*, a key effector required for intracellular survival, preserved robust tumor regression in NF2-SWN murine schwannoma models while improving safety and reducing systemic toxicity. To create a genetically defined and tractable platform, we generated two attenuated strains—AST101 and AST101-Δ*spiC*—which retain key mutations present in VNP20009 but lack ill-characterized background mutations. In the syngeneic NF2-SWN mouse schwannoma model, both strains significantly suppressed tumor growth compared to PBS. Collectively, these findings support the development of rationally engineered *Salmonella* Typhimurium strains with enhanced safety and preserved antitumor efficacy.

**IMPORTANCE:**

Given long-standing safety concerns surrounding the therapeutic use of live bacteria, we constructed a ΔspiC mutant of VNP20009 and demonstrated that it provides a markedly improved safety profile while retaining antitumor efficacy in NF2-related schwannomatosis mouse schwannoma models. In addition, we created two genetically defined *Salmonella* Typhimurium strains, AST01 and AST01-ΔspiC, which incorporate the key-targeted mutations found in VNP20009 and VNP20009-ΔspiC, respectively. These engineered strains offer a well-defined genetic background, enabling precise investigation of the bacterial traits responsible for *Salmonella* Typhimurium-mediated tumor control and thus further improvement of attenuated strains optimized for bacteriotherapy of neoplasms.

## INTRODUCTION

The first published report of bacteriotherapy for cancer dates back to 1868, when Wilhelm Busch used *Streptococcus pyogenes* to treat cancer. In 1891, William Coley, a surgeon in the United States, began using extracts of *Streptococcus pyogenes*—“Coley’s toxin”—and reported efficacy in patients with inoperable cancers (1–4). More than a century later, as our understanding of the tumor microenvironment (TME) deepens and systems biology expands our ability to engineer therapeutics, bacteriotherapy remains an actively investigated platform for cancer treatment. The bacterial strains used span attenuated pathogens and commensals, gram-negative and gram-positive species, live or inactivated organisms, and intracellular or extracellular lifestyles. These biologics share several advantageous features, including the ability to activate host immune pathways, susceptibility to a broad range of antibiotics, and well-defined genetics (5).

Certain bacteria, such as attenuated *Salmonella (S*.) Typhimurium, are well suited as vectors for tumor targeting because they are facultative anaerobes that preferentially colonize the hypoxic regions of solid tumors, enabling selective tumor localization while limiting host toxicity (6–9). VNP20009 is a highly mutagenized strain of *S*. Typhimurium derived from the wild-type strain 14028 (10–13). It was engineered to carry two key modifications: an *msbB* mutation, which produces lipopolysaccharide (LPS) lacking the myristic acid moiety of lipid A that reduces the risk of septic shock, and a *purI* mutation, which confers purine auxotrophy and restricts bacterial growth to purine-rich environments such as tumors (14, 15). Together, these features likely contribute to VNP20009 attenuation and tumor targeting. We previously reported that schwannomas are amenable to bacterial therapy, with intratumoral injection of VNP20009 controlling tumor growth and inducing host antitumor immune responses in preclinical NF2-related schwannomatosis (NF2-SWN) mouse models (12, 13, 16). However, because heavy ultraviolet (UV) light and chemical mutagenesis were used during the construction of VNP20009 (10), one cannot be sure which genetic lesions are responsible for its attenuation and efficacy as an anti-schwannoma therapeutic agent.

Schwannomas are benign peripheral nerve sheath tumors that can cause debilitating gain- and loss-of-function neurological deficits, including severe pain, hearing loss, tinnitus, motor loss, and balance disturbances (17, 18). These tumors occur in the genetic disorders NF2-related schwannomatosis (NF2-SWN; formerly neurofibromatosis type 2) and non–NF2-related schwannomatosis (previously NF3), and may also arise sporadically (then termed sporadic schwannoma) (19, 20). Conventional cancer therapies have limited efficacy against these slow-growing benign neoplasms. Surgical resection, the current standard of care, is non-curative, carries substantial risk of neurological injury, and is often impractical due to anatomical considerations (21, 22). Because of their hypoxia-rich regions, unique immune microenvironment, and low mitotic index (23), schwannomas create a favorable niche for the proliferation and antitumor activity of VNP20009, highlighting its potential clinical utility for benign neoplasms (16). Notably, no clinical trials to date have evaluated bacterial therapy for any benign tumor.

Despite extensive preclinical evidence supporting bacterial cancer therapy, clinical translation has lagged behind, and the number of clinical trials remains limited. Phase I trials with engineered *Salmonella* strains were disappointing, likely due to inadequate tumor targeting and systemic toxicity (24, 25). Consequently, multiple bacterial species have since been engineered to improve safety and tumor specificity by creating more defined, targeted delivery vehicles (26–31).

The molecular basis for the pathogenicity of *Salmonella* species has been studied for over a century in part because of its remarkable host-specificity as well as its impressive repertoire of virulence factors. Host specificity has been recently recognized to be associated with the elaboration of toxins, capsules, and effectors that target mammalian cellular machinery as well as the innate immune system (32–34). As facultative intracellular pathogens, all *Salmonella* species can actively invade (i.e., penetrate or enter) eukaryotic cells and survive within cytoplasmic endosomal compartments. Occupying this intracellular niche is largely driven by an organelle called the type III secretion system (T3SS), which is a nanomachine that can inject proteins (effectors) through both bacterial and eukaryotic membranes. *S*. Typhimurium expresses two T6SSs, encoded by chromosomal pathogenicity islands SPI-1 and SPI-2. In general, it is thought that the effectors injected by SPI-1 are responsible for active invasion of epithelial cells, while those injected by SPI-2 are involved in survival within professional phagocytic cells such as macrophages (35–38). Accordingly, mutations in genes such as *SipB* (encoded by SPI-1) or *SpiC* (encoded by SPI-2) can dramatically reduce the virulence of *S*. Typhimurium in appropriate animal models. Understanding how SPI-1 and SPI-2 encoded gene products affect bacterial immunotherapy of neoplasm in the context of an efficacious strain such as VNP20009 has not been systematically explored previously.

In the present study, we targeted bacterial loci involved in epithelial host cell invasion and survival within macrophages to develop VNP20009 derivatives with optimized safety profiles. Specifically, we deleted T3SS components of *Salmonella* pathogenicity island SPI-1 and SPI-2 and evaluated the resulting strains in NF2-SWN murine models. Our data show that deletion of the SPI-1 gene *sipB* only partially affects VNP20009 antitumor efficacy *in vivo*. In contrast, deletion of the SPI-2 structural component *spiC* (strain VNP20009-Δ*spiC*) preserved robust tumor growth control while improving safety, highlighting its translational potential. Recognizing that VNP20009 carries numerous uncharacterized mutations, we also engineered two *S*. Typhimurium “blueprint” strains on a genetically defined background—AST01 (carrying *msbB* and *purI mutations*) and AST01-Δ*spiC* (additionally carrying Δ*spiC*). Their antitumor effects in the syngeneic NF2-SWN mouse model were comparable to those of VNP20009 and VNP20009-Δ*spiC*, respectively. These findings enhance our understanding of bacterial determinants of tumor control and support the translation of newly defined attenuated *S*. Typhimurium strains toward clinical application.

## RESULTS

### Engineering *Salmonella* Typhimurium for enhanced binding and invasion of NF2-SWN cells

**Fig 1:**
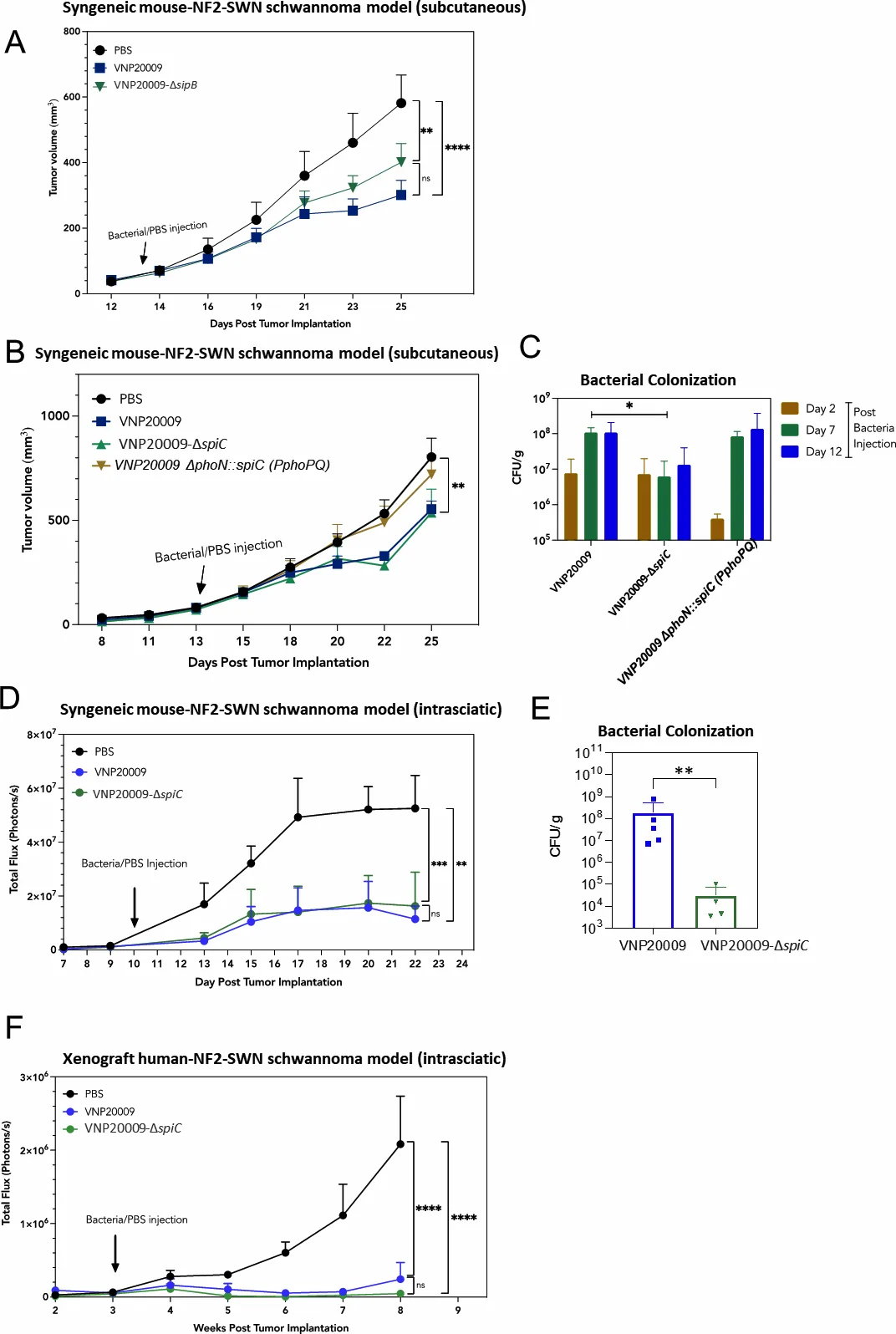
Evaluation of tumor control by VNP20009 SPI-1 and SPI-2 mutant strains in multiple tumor mouse models. (**A and B**) Tumor growth curves of 08031-9 schwannomas in FVB/N mice treated with VNP20009-Δ*sipB* (**A**), VNP20009-Δ*spiC or spiC*-overexpressing VNP20009 strain (**B**), or VNP20009, compared with PBS controls (*n* = 8/group). (**C**) Tumor-associated bacterial burden of VNP20009, VNP20009-Δ*spiC*, and *spiC*-overexpressing VNP20009 strains at days 2, 7, and 12, quantified as CFU/g tissue (*n* = 3/group). (**D and F**) Tumor growth of intrasciatic 08031-9 (**D**) and HEI-193 (**F**) schwannomas in FVB/N and nude mice, respectively, following treatment with VNP20009-Δ*spiC* or VNP20009 compared with PBS (*n* = 5/group). (**E**) Tumor-associated bacterial burden of VNP20009 or VNP20009-Δ*spiC* at day 22 in the intrasciatic syngeneic model (*n* = 5/group). For all panels, data represent mean ± SEM. Tumor volumes were analyzed using repeated-measures two-way ANOVA with Tukey’s test. Bacterial CFU data were analyzed using one-way ANOVA with Dunnett’s test (**C**) or Mann–Whitney test (**E**). *P < 0.05, **P < 0.01, ***P < 0.001, ****P < 0.0001, and ns, non-significant.

Despite robust preclinical data supporting VNP20009 as an antitumor agent in a variety of tumor models (11–13, 16, 39, 40), clinical trials of VNP20009 for metastatic malignant melanoma (and in one individual with renal cell carcinoma) have been disappointing (24, 41). In an effort to improve the safety profile of VNP20009, we investigated if critical genes in the two T3SSs of *S*. Typhimurium were required for its antitumor therapeutic potency. To disrupt SPI-1 T3SS function, we generated an in-frame deletion of *sipB* (Δ*sipB*), a core component of the SPI-1 translocon, the portion of the machine that forms the pore in the host cell membrane upon which it docks prior to delivering proteins into host cells (42). The Δ*sipB* mutant (VNP20009-Δ*sipB*) exhibited no detectable defects in growth when cultured in rich media such as LB (Fig. S1A) and was therefore evaluated for antitumor activity using a syngeneic NF2-SWN mouse model (16, 43). NF2-deficient murine schwannoma cells were implanted subcutaneously into FVB/N mice, followed by intratumoral (i.t.) administration of VNP20009, VNP20009-Δ*sipB*, or PBS. Our data showed that both VNP20009 and the VNP20009-Δ*sipB* strain significantly suppressed tumor progression relative to the PBS control (Fig. 1A). Tumor divergence was evident by day 10 post-injection, with VNP20009-treated mice demonstrating the strongest tumor control. Although VNP20009 consistently produced smaller tumor volumes than VNP20009-Δ*sipB* at all time points from day 8 post-injection onward, this difference did not reach statistical significance. By the study endpoint, VNP20009 reduced tumor size by approximately 2-fold relative to PBS, whereas VNP20009-Δ*sipB* achieved approximately a 1.5-fold reduction (Fig. 1A). These findings indicate that VNP20009-Δ*sipB* partially retains antitumor activity despite disruption of SPI-1 T3SS function.

To further dissect the role of *sipB* in bacterial interaction with tumor cells, we performed gentamicin protection assays to quantify binding and invasion of *Salmonella* strains in relevant mammalian cell lines. Caco-2 epithelial cells, RAW264.7 macrophages, and HEI-193 human NF2-SWN–derived cells were exposed to either *S*. Typhimurium 14028 (parent strain of VNP20009), VNP20009, or VNP20009-Δ*sipB*. After killing of extracellular bacteria by gentamicin treatment, intracellular bacteria were recovered 1 h post-infection. Across all tested cell lines, deletion of *sipB* did not alter invasion compared to VNP20009 (Fig. S1B), indicating that disruption of SPI-1 T3SS does not impair the baseline ability of VNP20009 *Salmonella* to interact with these cells under the tested conditions. Although invasion activity associated with the SPI-1 can vary depending on growth conditions, we did not pursue modifying these *in vitro* invasion assays further to reveal any potential correlation with what was, at best, only a minor reduction in efficacy of the Δ*sipB* mutant of VNP20009 compared to VNP20009.

Collectively, these findings demonstrate that modulation of SPI-1 T3SS components may influence *in vivo* tumor control, but the invasion assays we performed do not reliably predict the potency of *Salmonella*-mediated antitumor activity within the complex tumor microenvironment.

### Tumor-suppressive effects of the VNP20009-Δ*spiC* mutant in syngeneic and xenograft NF2-SWN mouse models

An overriding goal of our work is to engineer an attenuated *S*. Typhimurium strain with similar or superior antitumor efficacy to VNP20009, while exhibiting an improved toxicity profile. SpiC, an effector encoded by the SPI-2 locus and a component of the type III secretion system (T3SS), is required for secretion of T3SS-associated proteins (44). Prior studies demonstrated that deletion of *spiC* results in markedly decreased colonization of intestinal sites in a murine typhoid fever model (45).

Therefore, we generated a *spiC* deletion mutant of VNP20009 (hereafter, VNP20009-Δ*spiC*) and confirmed that loss of *spiC* does not compromise bacterial fitness in standard LB media (Fig. S1A). Similar to our results with the *sipB* mutant strain, deletion of *spiC* did not influence the invasion of the mammalian cell lines tested, including Caco-2, RAW264.7, and HEI-193 under the conditions tested (Fig. S1B). These findings are consistent with previous reports showing that Δ*spiC* mutants exhibit unaltered invasion capacity (45, 46).

To further investigate the effect of altered *spiC* expression on antitumor activity, we constructed a spiC-overexpressing VNP20009 mutant by integrating *spiC* into the *phoN* locus under control of the P*phoPQ* promoter (VNP20009 Δ*phoN::spiC* (P*phoPQ*)). The P*phoPQ* promoter is known to drive strong gene expression *in vivo* (47) and is activated by low pH and reduced magnesium levels *in vitro* (48, 49). As expected, qPCR analysis showed that the spiC-overexpressing strain cultured under low-magnesium minimal media conditions exhibited markedly higher levels of *spiC* transcripts compared with the parental VNP20009 strain grown under identical conditions (Fig. S2).

We next assessed the antitumor efficacy and intratumoral colonization profiles of VNP20009-Δ*spiC* and the *spiC*-overexpressing strain in the immunocompetent syngeneic NF2-SWN mouse model. Mice bearing subcutaneous (s.c.) 08031-9 NF2-deficient schwannoma tumors (~150 mm³) received i.t. injections of VNP20009, VNP20009-Δ*spiC*, the *spiC*-overexpressing strain, or PBS. Consistent with prior reports (12, 13, 16), VNP20009 induced robust tumor suppression within approximately 10 days. VNP20009-Δ*spiC* maintained equivalent tumor-suppressive activity to VNP20009, and both strains significantly reduced tumor growth relative to PBS control (Fig. 1B). In contrast, overexpression of *spiC* abrogated tumor growth control, indicating that precise regulation of *spiC* expression is critical for antitumor activity under these conditions. Analysis of bacterial burdens within tumors revealed that VNP20009-Δ*spiC* exhibited substantially reduced intratumoral persistence by days 7 and 12 when compared with VNP20009 and the *spiC*-overexpressing strain (Fig. 1C). These data suggest that a lower colonization threshold of VNP20009-Δ*spiC* bacteria is sufficient to achieve antitumor efficacy comparable to the higher intratumoral levels attained by the parental VNP20009.

To confirm these observations and establish broader applicability, we evaluated the antitumor efficacy of the VNP20009-Δ*spiC* strain in both orthotopic xenograft human NF2-SWN and syngeneic mouse NF2-SWN intra-sciatic schwannoma models. Luciferase-expressing HEI-193FC (human NF2-SWN schwannoma) or 08031-9FC (mouse NF2-SWN schwannoma) cells were implanted into the sciatic nerves of nu/nu mice (xenograft) and FVB/N mice (syngeneic), respectively (50–52). Once schwannoma growth was clearly established, tumors were injected i.t. with ~10^4^ CFU bacteria (VNP20009 and VNP20009-Δ*spiC*) or PBS. Tumor growth was monitored by *in vivo* bioluminescence imaging weekly (xenograft model) or every two days (syngeneic model). In both models, tumor growth profiles diverged between bacterial-treated and PBS-treated groups as early as day 7 post-treatment (Fig. 1D and F). Both VNP20009 and VNP20009-Δ*spiC* significantly suppressed tumor progression compared with PBS control groups. Remarkably, in the xenograft model, three of the five mice treated with VNP20009 and four of the five mice treated with VNP20009-Δ*spiC* exhibited complete absence of detectable tumor signals by the end of the study period. Examination of bacterial burden in tumors from syngeneic mice at day 22 post-implantation revealed ~2 × 10^8^ CFU/g of VNP20009, compared with only ~3 × 10^4^ CFU/g of VNP20009-Δ*spiC* (Fig. 1E). Together, these findings demonstrate that VNP20009-Δ*spiC* retains potent antitumor efficacy despite markedly reduced intratumoral colonization, suggesting a therapeutic window in which tumor control is preserved without high bacterial burdens that drive toxicity.

### Toxicity and biodistribution of the attenuated VNP20009-Δ*spiC* strain in FVB/N mice

Because *in vivo* toxicity remains a major barrier to the clinical translation of VNP20009-based cancer therapy, we conducted a biosafety assessment comparing VNP20009 with its Δ*spiC* derivative in immunocompetent FVB/N mice. In an initial pilot study, we injected groups of mice (*N* = 3 per group) intraperitoneally (i.p.) with PBS or increasing doses of VNP20009 or VNP20009-Δ*spiC*—2 × 10^5^ CFU (low), 2 × 10^6^ CFU (medium), and 2 × 10^7^ CFU (high). Sickness behavior and body weight were monitored daily, and organ inflammation was assessed by liver and spleen weight at sacrifice on day 11. Survival outcomes showed that mice receiving low or medium doses of either VNP20009 or VNP20009-Δ*spiC* displayed 100% survival, indistinguishable from PBS controls (Fig. S3A). However, high-dose VNP20009 (2 × 10^7^ CFU) resulted in a marked reduction in survival, with only 33% of mice surviving to day 11. In contrast, all mice injected with the same dose of VNP20009-Δ*spiC* survived to the study endpoint. Based on these data, we calculated the intraperitoneal LD_50_₀ of VNP20009 in FVB/N mice to be approximately 2 × 10^7^ CFU. Body weight measurements did not reveal differences between mice injected with PBS or 2 × 10^5^ CFU of either bacterial strain (Fig. S3B). At the 2 × 10^6^ CFU dose, mice receiving VNP20009-Δ*spiC* exhibited a transient decrease in body weight but recovered fully within 24 h. In contrast, mice treated with 2 × 10^6^ CFU VNP20009 experienced progressive weight loss, reaching approximately 10% below baseline by day 11 (Fig. S3C). Analysis of terminal organ weights showed no difference between PBS- and VNP20009-Δ*spiC*-injected mice at any dose. In contrast, mice injected with low or medium doses of VNP20009 displayed significantly enlarged livers and spleens, consistent with systemic inflammation (Fig. S3D). These results indicate that genetic ablation of *spiC* attenuates systemic toxicity relative to the parental VNP20009 strain in immunocompetent FVB/N mice.

**Fig 2:**
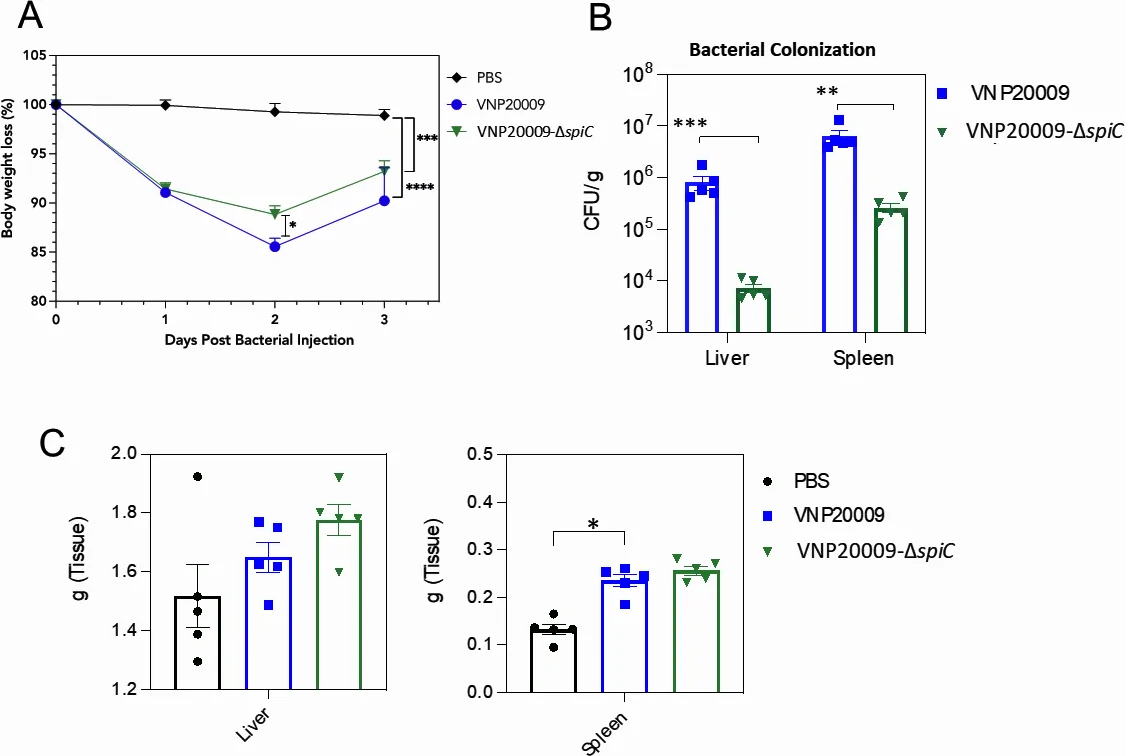
Early systemic toxicity and biodistribution of VNP20009-Δ*spiC* in FVB/N mice. (**A**) Body weight changes over 3 days following i.p. injection of PBS or 2 × 10^7^ CFU of VNP20009 or VNP20009-Δ*spiC* (*n* = 5/group). (**B**) Bacterial burden in liver and spleen at day 3 post-injection was quantified as CFU/g tissue (*n* = 5/group). (**C**) Liver and spleen weights at day 3 post-injection compared with PBS controls (*n* = 5/group). For all panels, data represent mean ± SEM. Statistical analyses were performed using repeated-measures two-way ANOVA with Tukey’s test (**A**), paired *t*-tests (**B**), or one-way ANOVA with Tukey’s test (**C**). *P < 0.05, **P < 0.01, ***P < 0.001, and ****P < 0.0001.

To investigate whether differences in bacterial colonization underlie these different toxicity profiles, we examined bacterial burdens in the spleen and liver following systemic i.p. challenge. Because 66% of animals injected with 2 × 10^7^ CFU VNP20009 were dead or moribund by day 4 in our pilot study (Fig. S3A), we selected day 3 post-injection as an early endpoint. Mice received i.p. injections of PBS, VNP20009 (2 × 10^7^ CFU), or VNP20009-Δ*spiC* (2 × 10^7^ CFU). Both bacterial strains caused immediate weight loss within 24 h. Thereafter, mice in both groups showed partial recovery by day 3, with VNP20009-Δ*spiC*-treated mice demonstrating a more pronounced rebound compared to VNP20009-treated mice (Fig. 2A). Quantification of bacterial load revealed extensive colonization of systemic sites by VNP20009, with recovery of ~10^6^ CFU/g in liver and ~10^7^ CFU/g in spleen. The VNP20009-Δ*spiC* strain exhibited significantly reduced colonization in both organs at the same time point (Fig. 2B). Despite these striking differences in bacterial burden, no significant differences in liver or spleen weights (an indicator of inflammation) were detected at day 3 (Fig. 2C), suggesting that systemic tissue inflammation may manifest later during infection.

**Fig 3:**
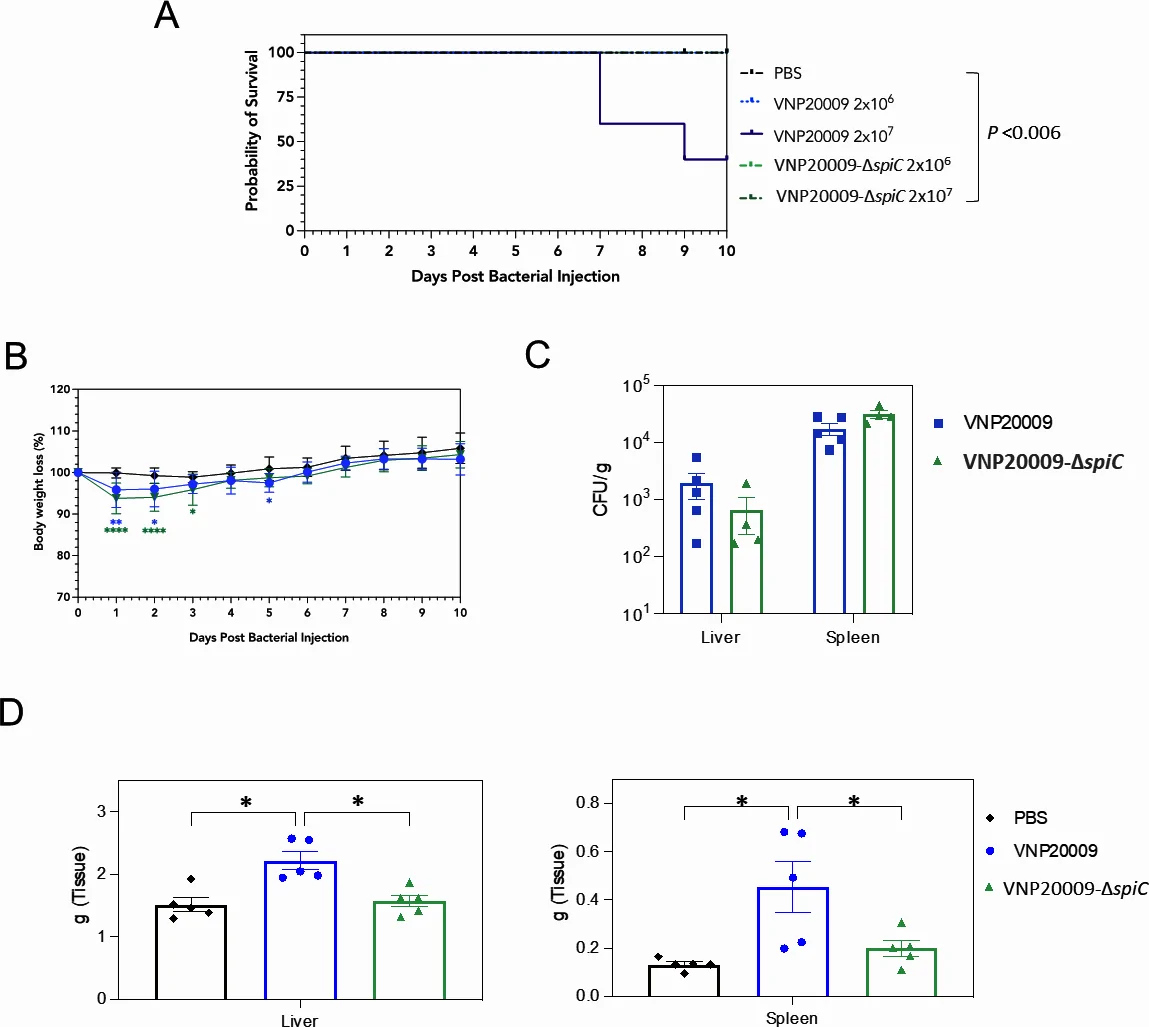
Extended systemic toxicity and survival analysis of VNP20009-Δ*spiC* in FVB/N mice. (**A**) Survival curves of mice injected i.p. with PBS, VNP20009, or VNP20009-Δ*spiC* (*n* = 5/group); the *P*-value shown refers to the comparison between the VNP20009 2 × 10^7^ CFU/mouse group and each of the other groups. (**B**) Body weight changes over 10 days following i.p. injection of PBS or 2 × 10^6^ CFU of VNP20009 or VNP20009-Δ*spiC* (*n* = 5/group). (**C**) Bacterial burden in liver and spleen at day 10 post-injection with 2 × 10^6^ CFU of VNP20009 or VNP20009-Δ*spiC*, quantified as CFU/g tissue (*n* = 5/group). (**D**) Liver and spleen weights at day 10 post-injection compared with PBS controls (*n* = 5/group). For all panels, data represent mean ± SEM. Statistical analyses were performed using repeated-measures two-way ANOVA with Tukey’s test (**B**), one-way ANOVA with Tukey’s test (**C and D**), and the log-rank test (**A**). *P < 0.05.

To validate and extend these findings, we performed a larger-scale biosafety study (*n* = 5/group) assessing both survival and organ colonization at a later time point. FVB/N mice were injected i.p. with PBS or with 2 × 10^6^ or 2 × 10^7^ CFU of VNP20009 or VNP20009-Δ*spiC*. Consistent with the pilot experiment, no mortality occurred in mice receiving VNP20009-Δ*spiC* at either dose, whereas 2 × 10^7^ CFU of VNP20009 again resulted in 66% mortality, confirming the previously defined LD_50_ for this attenuated strain (Fig. 3A). Mice injected with 2 × 10^6^ CFU of either VNP20009 or VNP20009-Δ*spiC* displayed a transient drop in body weight within the first 48 h but recovered fully, with weights indistinguishable from PBS controls later in the observation period (Fig. 3B). At day 10 post-injection, liver and spleen were harvested for bacterial enumeration. Both strains exhibited substantially lower bacterial burdens at this late time point compared with day 3. VNP20009 reached ~2 × 10^3^ CFU/g in spleen and ~2 × 10^4^ CFU/g in liver, with comparable levels observed for VNP20009-Δ*spiC* (Fig. 3C), indicating that by day 10 systemic colonization of both strains converges. Assessment of tissue weight at day 10 revealed that mice injected with VNP20009 displayed significantly heavier livers and spleens than PBS controls, whereas tissues from VNP20009-Δ*spiC*-injected mice remained similar in weight to PBS-treated animals (Fig. 3D). Together, these data demonstrate that the VNP20009-Δ*spiC* shows markedly reduced systemic colonization, organ weight, and toxicity compared to VNP20009 while retaining efficacy, supporting its potential as a safer attenuated *Salmonella* platform for schwannoma treatment.

### Generation of genetically defined bacteriotherapy strains

VNP20009 is derived from *S*. Typhimurium strains whose development induced multiple rounds of UV and chemical mutagenesis, as well as targeted genetic modifications (53). Genomic sequencing by our group and others has revealed that, in addition to the intended modifications, VNP20009 harbors a large 108 kbp deletion and ~90 single-nucleotide polymorphisms (SNPs) distributed throughout the chromosome. The functional consequences of these background mutations on bacterial physiology, host interactions, and antitumor efficacy remain unknown (16, 53). The genetic complexity of VNP20009—while tolerable for initial clinical exploration—poses significant challenges for mechanistic studies, rational optimization, and regulatory advancement to expanded clinical Phase I trials. To address these limitations, we sought to build a genetically defined strain, retaining several known VNP20009 phenotypical traits while providing a controlled and precisely modifiable genetic framework for tumor-targeting optimization.

**Fig 4:**
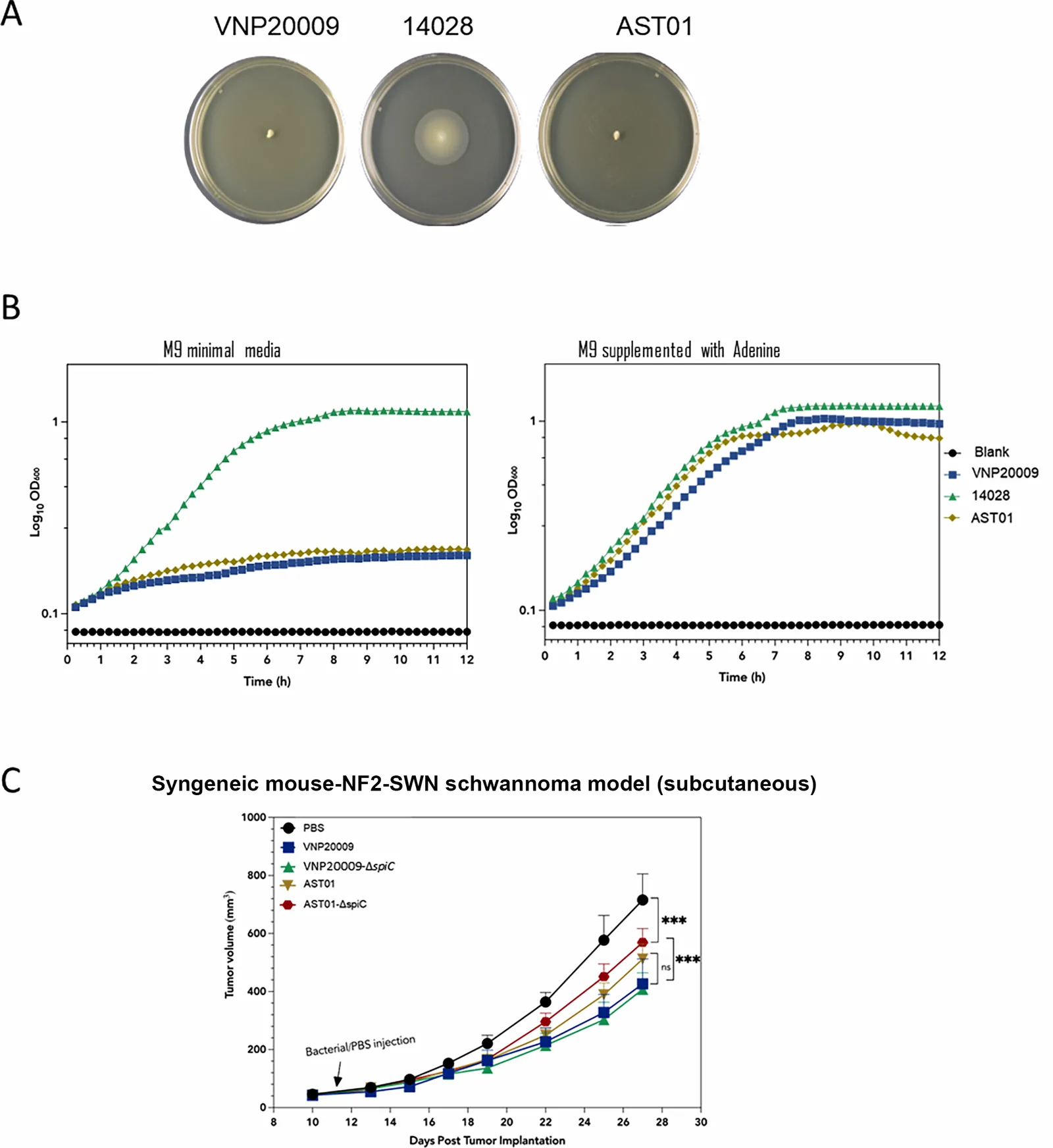
Phenotypic characterization of novel attenuated *S.* Typhimurium, strain AST-01, and its comparative antitumor activity in the mouse NF2-SWN schwannoma model. (**A**) Motility assay on 0.3% (wt/vol) agar plates following inoculation with 14028 (VNP20009 parental strain), VNP20009, and AST-01. (**B**) Growth of 14028, VNP20009 and AST-01 in M9 minimal medium with or without adenine was monitored over time by measuring optical density and depicted with an algorithmic scale (log_10_). Data are shown as the mean ± SEM of three technical replicates. (**C**) Growth of s.c. 08031-9 mouse NF2-deficient tumors in syngeneic FVB/N mice following i.t. injection of VNP20009, VNP20009-∆*spiC*, AST-01, and AST-01-∆*spiC*. Tumor volume was noninvasively monitored over time using direct caliper measurement (*n* = 8/group). Two-way repeated measures ANOVA with Tukey’s multiple comparisons test was used to compare groups. Data are shown as the mean ± SEM. ***P < 0.001; ns, non-significant.

Using the *S*. Typhimurium wild type 14028 background (the strain from which VNP20009 was derived), we introduced a set of in-frame deletions targeting known determinants of attenuation and tumor-selective growth: *msbB* (required for myristoylation of lipid A), *purI* (conferring purine auxotrophy), and the *motAB* operon (energizing flagellar motility but not flagella assembly). The resulting strain, designated AST01 (Δ*msbB*, Δ*purI*, and Δ*motAB*), incorporates these three defined mutations and phenotypic validation confirmed successful engineering of the intended traits. Deletion of *motAB* abolished motility, as evidenced by the lack of radial expansion in soft agar motility assays (Fig. 4A) but did not block production of potentially pro-inflammatory flagella (data not shown). Deletion of *purI* prevented growth in minimal media unless exogenous purine was supplied, establishing robust purine auxotrophy (Fig. 4B). In addition, to ensure enhanced safety, we incorporated a deletion of *spiC*, generating the final strain AST01-Δ*spiC (*Δ*msbB,* Δ*purI,* Δ*motAB*, and Δ*spiC*). We next evaluated the antitumor activity of these engineered strains in our syngeneic mouse-NF2-SWN schwannoma model (Fig. 4C). FVB/N mice bearing established s.c. tumors received i.t. injections of 10^4^ CFU of VNP20009, VNP20009-Δ*spiC*, AST01, AST01-Δ*spiC*, or PBS. As expected, VNP20009 and VNP20009-Δ*spiC* both demonstrated robust tumor control relative to PBS, consistent with their previously characterized antitumor activity. Notably, AST01 exhibited antitumor efficacy comparable to that of VNP20009, indicating that the defined genetic deletions did not compromise the strain’s therapeutic potential. AST01-Δ*spiC* also significantly delayed tumor growth compared with PBS, though the magnitude of tumor suppression was reduced relative to VNP20009 and VNP20009-Δ*spiC*. Consistent with earlier observations, VNP20009 and VNP20009-Δ*spiC* were indistinguishable in antitumor efficacy. The reduced activity of AST01-Δ*spiC* relative to Δ*spiC* in the VNP20009 background suggests that one or more of the unidentified SNPs or chromosomal deletions present in VNP20009 may compensate for the functional impact of *spiC* loss. This finding highlights the importance of using well-defined genetic backgrounds to dissect mechanisms inherent to the bacterial products that underlie bacterium-mediated anticancer effects. Together, these results demonstrate that AST01 preserves VNP20009 efficacy within a defined genetic background, eliminating unknown mutations and enabling precise mechanistic studies and rational engineering of *Salmonella*-mediated antitumor activity.

## DISCUSSION

Bacteria-mediated cancer therapy has attracted sustained interest due to its unique ability to selectively localize within and remodel the tumor microenvironment (54–56). Among the most extensively characterized platforms, the attenuated *S*. Typhimurium strain, VNP20009, has shown robust antitumor activity in multiple preclinical models (10–13, 16, 25, 57, 58) and therefore progressed to Phase I testing in patients with end-stage metastatic cancers (24). In those early trials, intravenous administration of VNP20009, while generally well tolerated, established dose-limiting toxicity (DLT), and no objective regressions were reported despite demonstration of tumor colonization in at least a subset of treated subjects (24). A separate, smaller clinical experience using TAPET-CD—VNP20009 engineered to express cytosine deaminase—similarly demonstrated acceptable safety and tumor colonization by bacteria in a subset of subjects, but limited antitumor activity (25). Together, these clinical experiences underscore two key limitations of the original VNP20009: (i) insufficient antitumor activity at tolerable systemic doses, and (ii) dose-limiting toxicity that potentially restricts therapeutic dosing, especially in settings where tumor control requires broader or repeated exposure to the bacterial product.

In this work, we sought to engineer next-generation bacteriotherapy strains that preserve the antitumor efficacy of VNP20009 while improving tumor specificity and safety. Our initial approach focused on the SPI-1 T3SS, which mediates invasion of non-phagocytic host cells (59). Deletion of *sipB* only modestly reduced antitumor efficacy in the syngeneic mouse NF2-SWN schwannoma model, indicating that disruption of a single invasion component encoded by SPI-1 is insufficient to meaningfully alter tumor specificity or efficacy. These findings are consistent with the multifactorial nature of *Salmonella*–tumor interactions, where bacterial invasion, cytokine induction, macrophage activation, nutrient deprivation, and hypoxic niche occupancy all contribute to therapeutic effects (57, 60–62).

Macrophages within the tumor microenvironment are increasingly recognized as critical mediators of bacterial anticancer responses (63). Prior work, including our own, has demonstrated that *S*. Typhimurium can reprogram tumor-associated macrophages (TAMs), shifting them from an M2 tumor-promoting phenotype toward an M1 tumoricidal state, and that bacterial survival within macrophages is important for effective tumor colonization and dissemination (45, 64, 65). Because the SPI-2 T3SS is essential for survival within phagosomes of macrophages (38, 66), we investigated whether modifying the SPI-2 locus could alter bacterial persistence and thereby influence antitumor efficacy or toxicity. Remarkably, deletion of *spiC* in the VNP20009 background produced a strain—VNP20009-Δ*spiC*—that exhibited antitumor activity equivalent to that of its parental strain—VNP20009—in both syngeneic mouse and xenograft human NF2-SWN schwannoma models, despite significantly reduced intratumoral colonization. These data reveal that systemic spread and high intratumoral bacterial burden are not prerequisites for therapeutic efficacy in this model and that VNP20009-Δ*spiC* retains sufficient tumor-associated activity to control tumor growth. Crucially, VNP20009-Δ*spiC* displayed markedly reduced toxicity in immunocompetent mice. Across multiple dosing regimens, VNP20009-Δ*spiC* caused less weight loss, lower mortality, and reduced splenic and hepatic inflammation when compared with VNP20009. Early after systemic exposure, VNP20009-Δ*spiC* colonized spleen and liver at significantly lower levels than VNP20009, supporting the notion that *spiC* contributes to systemic persistence and pathogenicity. Importantly, mice tolerated higher doses of VNP20009-Δ*spiC* than VNP20009 without morbidity, suggesting that VNP20009-Δ*spiC* could permit higher therapeutic dosing intratumorally—and potentially intravenously—than VNP20009. This has major translational implications for NF2-SWN, where tumors arise in proximity to critical neural structures and minimizing systemic and local toxicity is paramount.

Additional studies have reported that deletion of multiple SPI-2 genes in *S*. Typhimurium completely abrogated tumor growth suppression in a melanoma model, suggesting that the SPI-2 locus is essential for *S*. Typhimurium–mediated antitumor activity in that context (67). Notably, the same work also showed that loss of several SPI-2 components did not significantly attenuate virulence, a finding that contrasts sharply with our Δ*spiC* attenuation results and with the well-established role of SPI-2 in facilitating systemic dissemination and intracellular survival in murine infection models (45, 68–70). Several explanations may account for these discrepancies, including differences in tumor type, tumor microenvironment composition, immune context, intracellular trafficking pathways, and compensatory mutations present in the mutagenized VNP20009 genome. Indeed, prior observations that VNP20009 carries ~90 unintended chromosomal mutations raise the possibility that uncharacterized genetic interactions may influence SPI-2 function in poorly understood ways. Tumor-specific immune landscapes likely further shape bacterial behavior in ways that differ substantially between melanoma and NF2-SWN associated neoplasms.

The precise mechanisms through which VNP20009-Δ*spiC* exerts its antitumor effects are not yet elucidated. SpiC has been implicated in regulating flagellar synthesis, intracellular trafficking, and virulence factor expression (37). While VNP20009 is non-motile, its flagellar machinery remains intact, raising the possibility that altered *spiC* expression may modulate flagellin-driven immune activation pathways. Given our prior findings that VNP20009 promotes M1 macrophage polarization (16), we speculate that VNP20009-Δ*spiC* may further skew macrophage activation states or modulate cytokine networks in a manner that enhances tumoricidal responses while limiting systemic inflammation. Future studies will be required to dissect these mechanisms and to determine how Δ*spiC* affects interactions with macrophages, dendritic cells, and other immune populations within the tumor microenvironment.

Recognizing the genetic complexity of VNP20009 as a barrier to mechanistic insight, we constructed a genetically defined strain, AST01, that incorporates three precise mutations found in VNP20009 (i.e., Δ*msbB*, Δ*purI*, and Δ*motAB*). We also generated AST01-Δ*spiC*, which carries an additional deletion of *spiC* on the AST01 background. Thus, AST01-Δ*spiC* lacks the extensive background mutations present in VNP20009, the strain used in clinical testing. AST01 and AST01-Δ*spiC* recapitulated the antitumor efficacy observed with VNP20009 and VNP20009-Δ*spiC*, respectively, demonstrating that these defined mutations are sufficient to confer potent schwannoma growth suppression in the NF2-SWN schwannoma models. These findings confirm that genetically controlled strains can be constructed without sacrificing therapeutic potency, creating a tractable platform for mechanistic studies and future clinical translation.

In conclusion, we developed attenuated *S*. Typhimurium strains that retain strong antitumor efficacy in NF2-SWN schwannoma models while exhibiting improved safety profiles. VNP20009-Δ*spiC*, AST01, and AST01-Δ*spiC* provide genetically defined platforms for further bacteriotherapy development. Compared to their parental strains, VNP20009-Δ*spiC* and AST01-Δ*spiC* may enable safer administration and improved clinical feasibility for NF2-SWN associated schwannoma and potentially other tumor types. Future studies will define how these strains interact with the NF2-SWN schwannoma immune microenvironment and how they can be integrated into multimodal therapeutic strategies.

## MATERIALS AND METHODS

### Bacteria strains

*S*. Typhimurium and *Escherichia coli* strains used in this study are listed in Table S1. Bacteria were routinely grown aerobically at 37°C in LB broth or on LB agar plates. Antibiotics were added to the medium at the following concentrations: 0.1 mg/mL streptomycin and 0.1 mg/mL carbenicillin.

### Generation of *S*. Typhimurium strains

Plasmids used in this study are listed in Table S2. Deletions of *spiC* and *sipB* were generated as previously described (71). Briefly, ~600 bp flanking regions were amplified and cloned into the pRE107 suicide vector using Gibson Assembly (New England Biolabs). The *msbB* mutation was introduced by replicating the truncation present in VNP20009 (10, 72). For generation of the spiC-overexpressing strain, *spiC* was inserted into the phoN neutral site under control of the *phoPQ*-activated promoter (P*phoPQ*). Constructs were introduced into VNP20009 by bacterial conjugation, and chromosomal integrations were confirmed by PCR.

### Invasion assay

Invasion studies were performed as previously described (73). Overnight cultures were diluted 1:100 in LB and grown at 37°C to mid-log phase (OD_600_ ≈ 0.8). Bacteria were incubated with Caco-2 epithelial cells, RAW264.7 macrophages, or HEI-193 schwannoma cells at MOIs of 5:1, 10:1, and 50:1, respectively, for 1 h at 37°C (5% CO_2_). Cells were washed and treated with gentamicin (50 µg/mL, 30 min) to eliminate extracellular bacteria, then lysed with 1% Triton X-100. Serial dilutions were plated on LB agar, and CFUs were quantified.

### qPCR

RNA was extracted from cultured bacteria as previously described (74). Briefly, overnight cultures were diluted 1:100 in LB and grown at 37°C to mid-log phase (OD_600_ = 0.8–1.0). Cells were collected and resuspended in TRIzol (Thermo Fisher Scientific), and RNA was purified using the PureLink RNA Mini Kit (Invitrogen) with TURBO DNA-free treatment (Ambion). RNA concentration was measured by Qubit (Thermo Fisher Scientific). qRT-PCR was performed using KAPA SYBR Fast One-Step Master Mix on an Eppendorf Mastercycler RealPlex2, and expression levels were normalized to rpoD.

### Motility assay

Swarming assays were performed by inoculating 3 µL of normalized overnight bacterial culture onto LB medium containing 0.3% (wt/vol) agar and incubating at 30°C for 12 h. Spreading of bacterial growth away from the point of inoculation was interpreted as positive motility. The assay was performed in triplicate.

### Purine auxotrophy assay

For the purine auxotrophy assay, overnight cultures of *S*. Typhimurium were diluted and grown aerobically at 37°C in M9 minimal medium or M9 supplemented with 20 µg/mL adenine. Optical density at 600 nm (OD_600_) was measured using a Synergy plate reader.

### Animals

Nu/nu and FVB/N mice were maintained on a 12:12-h light–dark cycle with *ad libitum* access to food and water.

### Animal models

Schwannomas were generated by injecting HEI-193FC or 08031-9FC cells into the sciatic nerves of athymic nude or syngeneic FVB/N mice, respectively, as previously described (16, 50, 75). Two weeks (HEI-193FC) or 1 week (08031-9FC) after implantation, tumors were injected with 10^4^ CFU *S*. Typhimurium in 2 µL PBS. Tumor growth was monitored by bioluminescence imaging using an IVIS Spectrum system after d-luciferin administration. For the s.c. model, 08031-9 cells mixed 1:1 with Matrigel were grafted into FVB/N mice, and tumor volume was calculated as described (43). Once tumors reached 150 mm^3^, intratumoral injections were performed. Excised tumors were processed and plated on LB agar to determine bacterial load (CFU/g) as previously described (8).

### Biosafety animal study

VNP20009 and VNP20009-Δ*spiC* strains were administered by intraperitoneal injection to naïve FVB/N mice at various doses. Animals were monitored and weighed daily. On day 3 or day 11 post-injection, organs were removed, weighed aseptically, and homogenized in 5 mL of sterile PBS. The homogenates were plated onto modified LB agar and incubated for 24 h at 37°C. Colony-forming units (CFU) per gram of tissue were determined by counting colonies and normalizing to the weight of the recovered organ.

### Statistical analysis

Data are presented as mean ± SEM. Statistical tests are indicated in the figure legends. One- or two-way ANOVA was performed as appropriate, followed by *post hoc* multiple comparisons. Analyses were conducted using Prism (GraphPad).

## AFTER EPUB

*[This article was published on 10 August 2026 with errors in the abstract. The abstract was corrected in the current version, posted on 18 August 2026.]*

## SUPPLEMENTAL MATERIAL

Supplemental Material mbio.01275-26-s0001.docx Supplemental figures and tables.

ASM does not own the copyrights to Supplemental Material that may be linked to, or accessed through, an article. The authors have granted ASM a non-exclusive, world-wide license to publish the Supplemental Material files. Please contact the corresponding author directly for reuse.
