# Supplementary material for: Deletion of the Salmonella pathogenicity island 2 gene, spiC, in attenuated Salmonella Typhimurium VNP20009 optimizes its potential for bacterial schwannoma therapy
Source: mBio. 2026 Aug 10;17(9):e01275-26. doi: 10.1128/mbio.01275-26 (PMC13556218; doi:10.1128/mbio.01275-26)
Supplement: Supplementary file 1 [file mbio.01275-26-s0001.docx]

**Supplementary Figures**

**Figure S1. Deletion of SPI-1 and SPI-2 genes in *S.* Typhimurium VNP20009 does not impair growth in standard media or invasion of relevant cell lines.**
(**A**) Growth kinetics of VNP20009 and mutant derivatives monitored by OD₆₀₀ over time and plotted on a log₁₀ scale. Data represent mean ± SEM of three technical replicates.
(**B**) Invasion assays in HEI-193, Caco-2, and RAW264.7 cells infected at MOIs of 50, 10, and 5, respectively, with strain 14028, VNP20009, or mutant derivatives. After 1 hour at 37°C, extracellular bacteria were removed by gentamicin treatment, and intracellular bacteria were quantified by plating serial dilutions to determine CFU/mL at T₀. Results are expressed as percentage invasion (CFU/mL/T₀). Data represent mean ± SEM of ≥3 independent experiments.

Statistical analysis was performed using ordinary one-way ANOVA with Dunnett’s multiple comparisons test relative to VNP20009. * p<0.05, ** p<0.01.

**Figure S2. Validation of *spiC* overexpression under *in vitro* conditions.**
Relative RNA expression of *spiC* in VNP20009, VNP20009-Δ*spiC*, and VNP20009 *ΔphoN::spiC (*P*phoPQ)*. strains grown in M9 minimal medium supplemented with either 10 µM or 10 mM magnesium sulfate. Fold changes were normalized to the housekeeping gene *rpoD*, with *gmk* used as an additional control. Data represent mean ± SEM of two independent experiments. Statistical analysis was performed using two-way ANOVA with Dunnett’s multiple comparisons test relative to VNP20009. ND; No difference, **** p<0.0001.

**Figure S3. Preliminary toxicity assessment in FVB/N mice indicates reduced liver and spleen involvement following VNP20009-Δ*spiC* injection.**
(**A**) Survival curves of mice injected i.p. with PBS or increasing doses of VNP20009 or VNP20009-Δ*spiC* (n = 3/group). Survival was analyzed using the log-rank (Mantel–Cox) test.
(**B–C**) Body weight changes following i.p. injection of 2 × 10⁵ CFU (**B**) or 2 × 10⁶ CFU (**C**) of VNP20009 or VNP20009-Δ*spiC* compared with PBS (n = 3/group). Data represent percent body weight change over time. (**D**) Liver and spleen weights at day 11 post-injection with VNP20009 or VNP20009-Δ*spiC* compared with PBS controls (n = 3/group). For panels B–D, data represent mean ± SEM. Statistical analyses were performed using repeated-measures two-way ANOVA with Tukey’s test (B–C) and one-way ANOVA with Tukey’s test (D). Blue asterisks indicate significance relative to PBS; black asterisks indicate significance relative to VNP20009. * p<0.05., ** p<0.01.

# Supplementary Tables

# Supplementary Table 1. *S*. Typhimurium and *E. coli* strains used in this study

| Strain (used in manuscript) | Genotype | Reference |
| --- | --- | --- |
| 14028 | *S*. Typhimurium 14028 (CDC 6516-60) | ATCC 14028 |
| VNP20009 | *S*. Typhimurium YS1646 (Δ*msbB* Δ*purI*) | ATCC BAA-3199 |
| VNP20009-Δ*sipB* | VNP20009 Δ*sipB* (Sm^r^) | This study |
| VNP20009-Δ*spiC* | VNP20009 Δ*spiC* (Sm^r^) | This study |
| VNP20009 ΔphoN::spiC (PphoPQ) | VNP20009 Δ*phoN::spiC* (P*phoPQ*) | This study |
| AST101 | 14028 Δ*msbB* Δ*purI* Δ*motAB* | This study |
| AST101 Δ*spiC* | 14028 Δ*msbB* Δ*purI* Δ*motAB* Δ*spiC* | This study |
| DH5α λpir | F– endA1 hsdR17 (rK– mK+) supE44 thi-1 recA1 gyrA96 relA1 Δ(lacZYA-argF)U169 deoR nupG λpir | Grant et al., PNAS 1990^1^ |
| S17-1 λpir | thi pro hsdR– hsdM+ recA RP4-2-Tc::Mu-Km λpir | Simon et al., Nat. Biotechnol. 1983^2^ |

*Sm^r^: streptomycin resistance; Carb^r^: carbenicillin resistance.

**Supplementary Table 2**. Plasmids used in this study

| **Designation** | **Relevant Characteristics** | **Reference/source** |
| --- | --- | --- |
| pRE107 | ori(R6K), mobRP4, sacB1 (Carb^r^ ) | Edwards et al., Gene. 1998: 207(2):149-57.^3^ |
| p∆sipB | *sipB* flanking regions including first and last 15 nucleotides of *sipB* CDS cloned into pRE107; insertion of p∆sipB into the VNP20009 chromosome disrupts the *sipB* gene | This Study |
| p∆spiC | *spiC* flanking regions including first and last 15 nucleotides of *spiC* CDS cloned into pRE107; insertion of p∆spiC into the VNP20009 chromosome disrupts the *spiC* gene | This Study |
| pSpiC-OE (pTAc) | *spiC* CDS under the tac promoter flanked by the *phoN* CDS flanking regions cloned into the pRE107; insertion of pSpiC-OE (pTac) into VNP20009 chromosome leads to overexpression of *spiC* gene | This Study |
| pSpiC-OE (pPhoPQ) | *spiC* CDS under the *phoPQ*-activated promoter flanked by the *phoN* CDS flanking regions cloned into the pRE107; insertion of pSpiC-OE (pPhoPQ) into VNP20009 chromosome leads to overexpression of *spiC* gene | This Study |
| p∆msbB | *msbB* first 552 nucleotides and 3’ flanking region cloned into the pRE107; insertion of p∆msbB into 14028 chromosome leads to gene truncation | This Study |
| p∆purI | *purI* (*purM*) flanking regions including first and last 15 nucleotides of *purI* CDS cloned into pRE107; insertion of p∆purI into the 14028 chromosome disrupts the *purI* gene | This Study |
| p∆motA | *motAB* flanking regions including first and last 15 nucleotides of *motAB* CDS cloned into pRE107; insertion of p∆motAB into the 14028 chromosome disrupts the *motAB* operon | This Study |

*Sm^r^: streptomycin resistance; Carb^r^: carbenicillin resistance.

1	Grant, S. G., Jessee, J., Bloom, F. R. & Hanahan, D. Differential plasmid rescue from transgenic mouse DNAs into Escherichia coli methylation-restriction mutants. *Proc Natl Acad Sci U S A* **87**, 4645-4649, doi:10.1073/pnas.87.12.4645 (1990).

2	Simon, R., Priefer, U. & Pühler, A. A broad host range mobilization system for in vivo genetic engineering: transposon mutagenesis in Gram-negative bacteria. Bio/Technology 1, 784–791, doi:10.1038/nbt1183-784 (1983).

3	Edwards, R. A., Keller, L. H. & Schifferli, D. M. Improved allelic exchange vectors and their use to analyze 987P fimbria gene expression. *Gene* **207**, 149-157, doi:10.1016/s0378-1119(97)00619-7 (1998).
